# Heterogeneous Host Susceptibility Enhances Prevalence of Mixed-Genotype Micro-Parasite Infections

**DOI:** 10.1371/journal.pcbi.1002097

**Published:** 2011-06-30

**Authors:** Wopke van der Werf, Lia Hemerik, Just M. Vlak, Mark P. Zwart

**Affiliations:** 1 Centre for Crop Systems Analysis, Wageningen University, Wageningen, The Netherlands; 2 Biometris, Wageningen University, Wageningen, The Netherlands; 3 Laboratory of Virology, Wageningen University, Wageningen, The Netherlands; 4 Instituto de Biología Molecular y Celular de Plantas, Consejo Superior de Investigaciones Científicas-UPV, Valencia, Spain; 5 Quantitative Veterinary Epidemiology Group, Wageningen University, Wageningen, The Netherlands; ETH Zurich, Switzerland

## Abstract

Dose response in micro-parasite infections is usually shallower than predicted by the independent action model, which assumes that each infectious unit has a probability of infection that is independent of the presence of other infectious units. Moreover, the prevalence of mixed-genotype infections was greater than predicted by this model. No probabilistic infection model has been proposed to account for the higher prevalence of mixed-genotype infections. We use model selection within a set of four alternative models to explain high prevalence of mixed-genotype infections in combination with a shallow dose response. These models contrast dependent versus independent action of micro-parasite infectious units, and homogeneous versus heterogeneous host susceptibility. We specifically consider a situation in which genome differences between genotypes are minimal, and highly unlikely to result in genotype-genotype interactions. Data on dose response and mixed-genotype infection prevalence were collected by challenging fifth instar *Spodoptera exigua* larvae with two genotypes of *Autographa californica* multicapsid nucleopolyhedrovirus (AcMNPV), differing only in a 100 bp PCR marker sequence. We show that an independent action model that includes heterogeneity in host susceptibility can explain both the shallow dose response and the high prevalence of mixed-genotype infections. Theoretical results indicate that variation in host susceptibility is inextricably linked to increased prevalence of mixed-genotype infections. We have shown, to our knowledge for the first time, how heterogeneity in host susceptibility affects mixed-genotype infection prevalence. No evidence was found that virions operate dependently. While it has been recognized that heterogeneity in host susceptibility must be included in models of micro-parasite transmission and epidemiology to account for dose response, here we show that heterogeneity in susceptibility is also a fundamental principle explaining patterns of pathogen genetic diversity among hosts in a population. This principle has potentially wide implications for the monitoring, modeling and management of infectious diseases.

## Author Summary

What elements are indispensable in the description of the most basic host-pathogen interactions? The simplest models of infection generally fail to predict how many host plants or animals will become infected, and which virus genotypes will be present in these infected hosts. These simple models of infection are the building blocks for more complicated models of epidemiology and disease dynamics and diversity, making it important to identify the reasons for failure. We developed four probabilistic models of infection incorporating different mechanisms that could potentially explain and overcome this failure. We obtained experimental data to test these models by exposing Lepidopteran larvae to different genotypes of an insect DNA virus, and determining which virus genotypes had infected them. The model which best described the data added only one element: variation in the susceptibility of individual caterpillars to the virus. Host variation in susceptibility is known to affect transmission of viruses between hosts, but here we show it is inextricably linked to infection biology and indispensable for understanding pathogen diversity in host populations.

## Introduction

Models of micro-parasitic infection and transmission have been instrumental to the study of infectious disease dynamics [1], [2]. The insights reaped from these models, together with the advent of an evolutionary biology framework, have revolutionized our understanding of infectious diseases, and impacted intervention and management strategies [3], [4]. A comparatively well tested aspect is how the rate of host infection is influenced by the density of infectious hosts [5], [6], or the concentration of micro-parasite infectious units [7]. If it is known how the rate of host infection changes, it is possible to predict dynamic behavior with simple epidemiological models [1]. Moreover, predictions of dose-response relationships can be extended to situations not readily measurable in the laboratory [8]. What is clear, however, is that the data generally do not support simple model predictions for dose-response relationships [7], [9], [10], [11], [12], [13]. It is not entirely clear what mechanisms are responsible for deviations from model predictions, but heterogeneity in host susceptibility to infection is often implicated as an explanatory factor.

An emerging area of concern for models of micro-parasite infection, where less model development has been conducted, is the occurrence of mixed-genotype infections. The extent to which mixed-genotype infections occur determine and constrain: (i) recombination between different micro-parasite genotypes, (ii) competition between genotypes at the within-host level, which may be an important determinant of virulence [14], and (iii) cooperation between different micro-parasite genotypes [15], [16]. In a previous study, we found that the frequency of mixed-genotype infections is not readily predictable in laboratory settings [13]. In nature, mixed-genotype infection of the same host is common for many micro-parasites, including baculoviruses [17], [18], [19]. What mechanisms are responsible for this diversity? Does this prevalence of diversity mean that many micro-parasite entities are needed to infect a host and cooperation is needed to overcome host resistance? Or does infection with one micro-parasite genotype or entity make infection by others easier, i.e. facilitation? These questions have not been widely studied, and little work has to our knowledge been conducted to develop a synthetic modeling framework to facilitate interpretation of empirical data on micro-parasite diversity in hosts. Here we develop such a framework. To confront alternative models in the framework with empirical data, we conducted experiments with an insect virus from the *Baculoviridae* family.

Baculoviruses are arthropod DNA viruses with large dsDNA genomes, and most are obligate host killers [20], [21]. Mixed-infections are highly relevant for understanding the ecology of natural baculovirus populations. Frequency-dependent selection of genotypes occurs [16], and there appear to be forms of facilitation between genotypes [16], [22]. Moreover, mixed-genotype infections appear to be highly relevant for determining the fitness and ecological impact of faster-acting genetically modified baculoviruses [23], [24], [25].

The simplest conceivable model of micro-parasite infection that makes predictions of both dose-response relationships and the frequency of mixed-genotype infections is based on the independent action hypothesis (IAH) [13], [26]. IAH states that each micro-parasite infectious unit (e.g., a single bacterium or virion) has the ability to infect the host on its own, and that each infectious unit can act independently, irrespective of the presence of other infectious units [26]. IAH in a homogeneous host population results in a dose response with a fixed shape [26], [27]. However, dose response experiments tend to result in a relationship that is at least somewhat shallower than predicted by IAH in a homogeneous host population [7], [9], [10], [11], [12], [13]. Some studies have therefore considered alternative models of infection: antagonistic dependent action or heterogeneous host susceptibility [7]. It is, however, difficult to differentiate between alternative models of infection based only on dose response data. Both the antagonistic dependent action and heterogeneous host susceptibility models predict shallow dose responses with only subtle differences [7], [9].

Another avenue to explore the validity of IAH has been to consider the frequency of mixed-genotype infections. By ‘mixed genotypes’ we refer to micro-parasite variants that can be distinguished at the genotypic level, but are virtually identical at the phenotypic level. We do not consider the effects of major phenotypic differences or genotype-genotype interactions between micro-parasite variants. Rather, we formulate general models that predict the occurrence of mixed-genotype infections when there are no interactions between micro-parasite genotypes in the infection process. The artificial genotypes we use in our experiments correspond well to the model assumptions, because these genotypes differ only by a 100 bp qPCR recognition sequence in a non-coding region [28], less than 0.1% of the genome. The only phenotypic difference we have observed between these genotypes is a minor difference in probability of infection [28], which can be accounted for in an infection model [13].

High frequencies of single-genotype infection have been reported when low micro-parasite doses are used, and interpreted as evidence for independent action [17], [29], [30]. However, even if there is dependence between micro-parasite infectious units, we could still expect to observe a high frequency of single-genotype infections at low doses. On the other hand, if there are antagonistic interactions between infectious units, than we could expect a low frequency of mixed-genotype infections over an extended range of doses including those causing substantial mortality. What is needed, therefore, is a mathematical model of infection that makes quantitative predictions of the frequency of mixed-genotype infections as a function of dose. We previously developed such a model of infection based on IAH. This model predicts the frequency of mixed-genotype infection based on host survival and the micro-parasite infection probability, assuming the latter to be constant in the host population [13]. In two out of six pathosystems tested, the frequency of mixed-genotype infections matched our model predictions. Dose response data are – in these instances – also similar to model predictions. However, in the remaining four pathosystems tested, the frequency of mixed-genotype infections was higher than predicted by IAH, and dose response was shallower. We therefore rejected this IAH-based model. Our data suggested that tests of IAH based on dose response and prevalence of mixed-genotype infection give congruent results [13].

These results, combined with the observation that dose response relationships tend to be shallower than predicted by IAH, suggest the IAH model in a homogeneous host population [13], [26] may not be generally applicable. It is therefore important to determine what model adaptations would result in better predictions. We studied the infection process in a pathosystem for which we previously rejected IAH: *Autographa californica* multicapsid nucleopolyhedrovirus (AcMNPV) in fifth instar larvae (L5) of the beet armyworm, *Spodoptera exigua* (Lepidoptera: Noctuidae) [13]. We formulated four probabilistic models of infection by relaxing one or both of two key assumptions of the standard IAH-based model for infection. The first assumption to be relaxed was the constancy of host susceptibility. The second assumption that we relaxed was the independence between pathogen entities. We empirically determined both the dose response and the frequency of mixed-genotype infections at different doses, and calibrated each of the four models. We could then identify which model best described the empirical results and reconciled a high frequency of dual genotype infections with a shallow dose response.

## Materials and Methods

### Ethics statement

All animals were handled in strict accordance with good animal practice as defined by the relevant national animal welfare bodies.

### Bioassays and quantitative real-time PCR

A droplet feeding bioassay was performed with newly molted (0–8 hours post head-capsule slippage) *S. exigua* L5, starved for 16 h, as described previously [13]. L5 were challenged with a 1∶1 occlusion body (OB) mixture of vPolhA and vPolhB bacmid-derived genotypes [28]. Note that OBs were separately amplified to avoid the possibility of co-occlusion of the different genotypes in single OBs or occlusion derived virus (ODV) [31]. Twenty-four larvae were taken as a non-virus control. The same number was infected per dose, with 10-fold dilutions ranging from 10^3^–10^9^ OBs/ml. As the number of larvae in a synchronized cohort was limited, the entire range was not taken in a single replicate, but a subset of ranges taken in each replicate. Mortality was recorded daily and dead larvae were collected and stored individually at −20°C. Subsequent OB purification, DNA isolation and quantitative real-time PCR (qPCR) were performed as described on a random subsample of infected larvae from the doses 10^5^, 10^6^, 10^7^, and 10^8^ [28].

### Baculovirus infection process and overview of the models

We first give our conceptual perspective of the baculovirus infection process, followed by an overview of the proposed infection models. The full process from ingestion of baculovirus OBs to infection can be divided into 5 main steps. Step 1: OBs are ingested by an insect larva. In our experimental setup, larvae, after a short period of starvation, drink individually a suspension of OBs. Step 2: OBs are degraded by the alkaline pH in the larval midgut, a process we refer to as OB dissolution. Step 3: Part of the liberated ODV (occlusion derived virus) bypasses the peritrophic membrane and enters an epithelial midgut cell. Subsequently, budded virus (BV) is secreted from the midgut cells into the interior tissues of the host (e.g. trachea, haemocytes and fat body). The successful outcome of the entire process so far described (steps 1–3) we will refer to as ‘penetration’ of the host. Step 4: The virus can then be further amplified in the host, a process we refer to as replication. Step 5: If the virus is amplified sufficiently, the host insect dies. Sufficiently amplified virus can be measured by qPCR. Virions which contribute to the entire process (steps 1–5) have ‘infected’ the host. Thus, we equate host infection with the occurrence of host death and PCR detection of the virus in the cadaver. There are therefore no separate measurements for penetration and infection. The qPCR assay does not detect viral genotypes in hosts that do not die after challenge with OBs, demonstrating that the assay does not detect virions which have only penetrated the host [13] (assuming such non-pathogenic penetration does occur).

To derive the models, we simplify the above five steps by distinguishing only two phases in the infection process: (i) penetration (steps one to three), and (ii) infection (steps four and five). Penetration entails the presence of the virus in the host, but we postulate that this will in itself not lead to host death or production of viral progeny, and (ii) infection: production of viral progeny and host death. Similarly, we will use the terms ‘mixed-genotype penetration’ and ‘mixed-genotype infection’ to indicate whether multiple viral genotypes have penetrated or infected the host.

**Figure 1:**
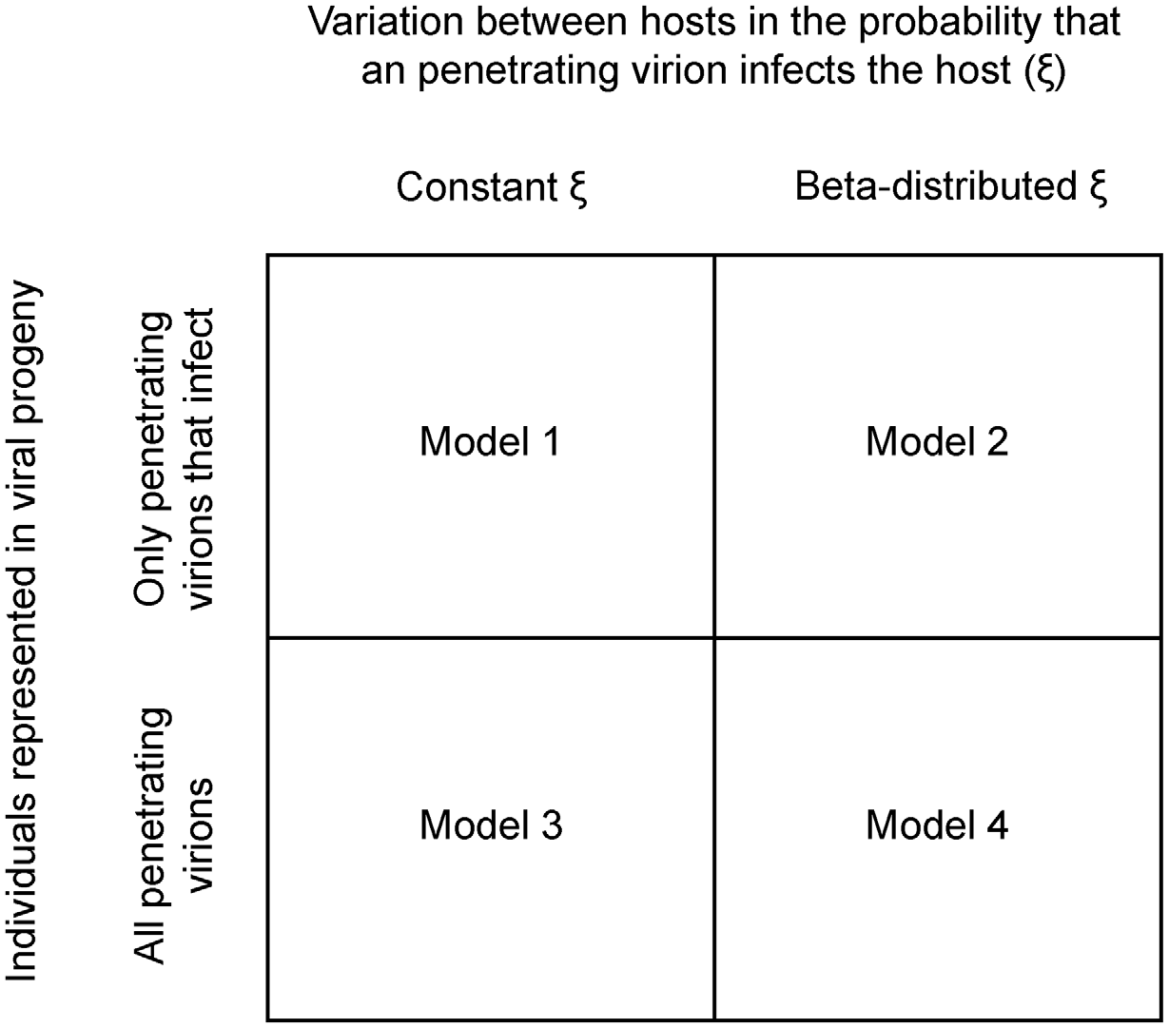
An overview of the four models postulated.

We consider four probabilistic models of this two-phase infection process (Figure 1). First, the virions must successfully bypass the midgut and reach interior host tissues (‘penetration’; up to step three of the infection process). Second, to cause host death, successfully penetrated virions should multiply in the host (‘infection’; steps four and five of the infection process). All the four models proposed consider only these two phases. We ignore variation in ingested dose because the effects of the measured variation in ingestion on dose-response were very minor for a similar experimental setup [11], [32]. We also ignore variation introduced by degradation of OBs, although there is undoubtedly variation in the number of virions (ODV) per OB [33]. L5 larvae are typically highly resistant and must therefore ingest large numbers of OBs to become infected, so the variation in individual OBs can be ignored. We do not incorporate any details of the viral replication phase, and simply focus on the outcome of this phase: does the host die, and if so, which pathogen genotypes are detectable in the cadaver?

For all four models, we assume that the virions in OBs (ODV) are ‘micro-parasite infectious units’, because they are released into the midgut lumen prior to infecting midgut cells, and therefore act independently of the OB [34]. For all four models the following holds: given a virus ODV population composed of *n*_A_ and *n*_B_ individuals of genotypes A and B, with penetration probabilities *p*_A_ and *p*_B_, the mean number of penetrators of each genotype is *λ*_A_ = *p*_A_ *n*_A_ and *λ*_B_ = *p*_B_ *n*_B_. A detailed derivation of the models is given after the model descriptions, in the materials and methods section entitled ‘Mathematical formulation of probability models’. The meaning of all symbols is given in Table 1.

**Table 1:** Symbols that pertain throughout the paper.

Symbol	Meaning
*n*_A_	Challenge number of individuals (virions) of genotype A
*n*_B_	Challenge number of individuals (virions) of genotype B
*n*	Challenge number of individuals (virions)
*p*_A_	Penetration probability for individuals of genotype A
*p*_B_	Penetration probability for individuals of genotype B
*p*	Penetration probability
*λ*_A_	Mean number of penetrating individuals of genotype A
*λ*_B_	Mean number of penetrating individuals of genotype B
*λ*	Mean number of penetrating individuals
*ξ*	Probability that a penetrating virion causes infection; fixed in models 1 & 3, a realization of *σ* in models 2 & 4.
*σ*	Probability that a penetrating virion causes infection; beta-distributed over hosts
*α*	Parameter of the beta distribution
*β*	Parameter of the beta distribution
Ω	The number of penetrating virions that infect the host
*ω*	A realization of Ω
*M*	Host mortality (proportion), equivalent to definition of host infection
*S*	Host survival
*ρ*	Probability of penetration and infection, equivalent to the product *ξ p*
*x_ρ_*(*n*)	Expected level of host mortality at dose *n*
*y_ρ_*(*n*)	Expected level of mixed genotype infections at dose *n*
*x*	Simplified notation for *x_ρ_*(*n*)
*y*	Simplified notation for *y_ρ_*(*n*)
*f*_A_	Frequency of genotype A in the inoculum (i.e., *n*_A_/*n*)
*f*_B_	Frequency of genotype B in the inoculum
*ϕ*	a real convex function
*x_i_*	Values of *x_ρ_*(*n*) within the domain of *ϕ*
*a_i_*	Weights of *x_i_*

### Model 1: Penetrating virions have a fixed probability of causing infection

The postulate that not all penetrating virions will cause infection is relevant because baculoviruses are known to cause sub-lethal infections [35], [36]. In the framework of model 1, sub-lethal infections would be considered as penetrating virions that do not cause infection, although perhaps remaining quiescent and below the detection threshold for a one-step PCR for viral genomic DNA in the host. The existence of sub-lethal infection supports the notion that penetration does not necessarily lead to death. The first model assumes: (1) independent action in host penetration, and (2) relaxes the assumption that penetration of the host by one or more virions will inevitably lead to infection (i.e., host death). Instead, we assume that each penetrating virion has a fixed probability *ξ* of causing infection (i.e. these virions have contributed to host death and are represented in the viral progeny).

This model, although incorporating two infection phases, is equal to the IAH model previously formulated [13], except that infection is now a two-phase process with success probability *ξ·p*, and the model therefore has a different parameterization than before. The definition of penetration, as proposed for model 1, allows virions to remain quiescent (not cause infection) after having penetrated the host, but virions still operate independently. This model is useful for illustrating that IAH does not specify the number of phases culminating in host infection: virions must, however, act independently during all phases involved. For model calibration, the product *ξ·p* must be estimated.

### Model 2: Penetrating virions have a beta-distributed probability of causing infection

Differences in host susceptibility may be important for understanding dose response relationships. This suggestion has been made for baculoviruses [11], [37] and a bacterial pathosystem [7], [9], [38]. However, the effects of variation in host susceptibility on the frequency of mixed-genotype infection have, to our knowledge, not been considered.

Model 2 is an extension of model 1. It relaxes the assumption of a fixed infection probability *ξ*, by postulating that the infection probability varies over hosts. Let *σ* be the stochastic variable for the infection probability and let *σ* follow a beta distribution over hosts. Each different realization of the stochastic variable *σ* applies to an individual insect host and all these realizations together represent the distribution of *ξ*. The beta distribution was chosen because of its versatility [39] and because there is a precedent for its use in describing variation in host susceptibility [11], [12]. The infection two beta distribution parameters (*α* and *β*) and the penetration probability *p* were separately identified from data

### Model 3: Penetrating virions have a fixed probability of causing infection and all penetrating virions are represented in the viral progeny

Models of dependent action can result in dose response relationships that are shallower than those predicted by IAH models with a fixed probability of infection [7], [9]. These models of dependent action are formulated using a synergy/antagonism parameter that appears as an exponent *k* on the micro-parasite dose:
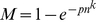
(1)


Where *M* is mortality in the host population, *p* is a parameter for infection probability, and *n* is the parasite dose per host. One mechanistic interpretation of this model is that the infection probability per micro-parasite infectious unit is modulated by parasite dose according to:
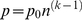
(2)where *p*_0_ is the probability of infection per micro-parasite at a dose of 1 micro-parasite per host. While this model is effective in modulating dose response, it does not change the genotype frequencies in cadavers at a given dose, because the infection probability is fixed given the dose, and micro-parasite infectious units are still acting independently at the given dose. In other words: this model is not useful for modeling genotype frequency response to dose that differs from the IAH model with a fixed probability of infection (i.e., Model 1).

Instead, we propose a two stage approach with a straightforward mechanistic interpretation. First, we postulate that parasites of two identifiably different genotypes (but same phenotype) penetrate the host independently, using the standard IAH assumptions. Next, we postulate that each penetrated micro-parasite attempts to infect the host (cause a systemic response), again using IAH. Then, we postulate that with the breakdown of host defense upon infection by the first successful micro-parasite, all penetrated micro-parasites can multiply, such that their progeny can be detected in the host. This set of assumptions can in principle explain an increased frequency of mixed genotype infections in cadavers, but the question is whether it is supported by data.

A two-stage model of the infection is empirically justified by the existence of so-called *latent infections* with baculoviruses. Latent baculovirus infections are vertically transmitted sub-lethal infections. A latent virus can be activated when host defenses have been weakened by biotic or abiotic stress, such as infection of the host with another baculovirus [40], [41]. This observation suggests that the mechanisms of dependence we propose is plausible, given that the breaking of host defenses by one virus allows another virus to be represented in the viral progeny subsequently generated.

Model 3 assumes – like models 1 and 2 – that each penetrating virion has a probability *ξ* of causing infection. As in model 1, we again assume there is no variation in susceptibility in the host population. However, unlike models 1 and 2, we here assume that all penetrating virions will be represented in the viral progeny if any one penetrating virion infects the host. In this model, different combinations of *ξ* and *p* values will render different results; although the dose response is determined by their product, mixed-genotype infection at a given level of mortality will increase with the number of penetrating virions (high *p*). The parameters *ξ* and *p* must both be estimated from the data.

### Model 4: Penetrating virions have a beta-distributed probability of causing infection and all penetrating virions are represented in the viral progeny in infected hosts

This model is a further extension of model 3. As in model 2, the probability of infection (*ξ*) is for each individual host a (different) realization of a stochastic variable *σ*, which follows a beta distribution over hosts. This model thus combines host heterogeneity in susceptibility with true dependency with respect to the infection process. For the calibration of this model, three parameters must be estimated: *p*, *α* and *β*.

### Mathematical formulation of probability models

**Figure 2:**
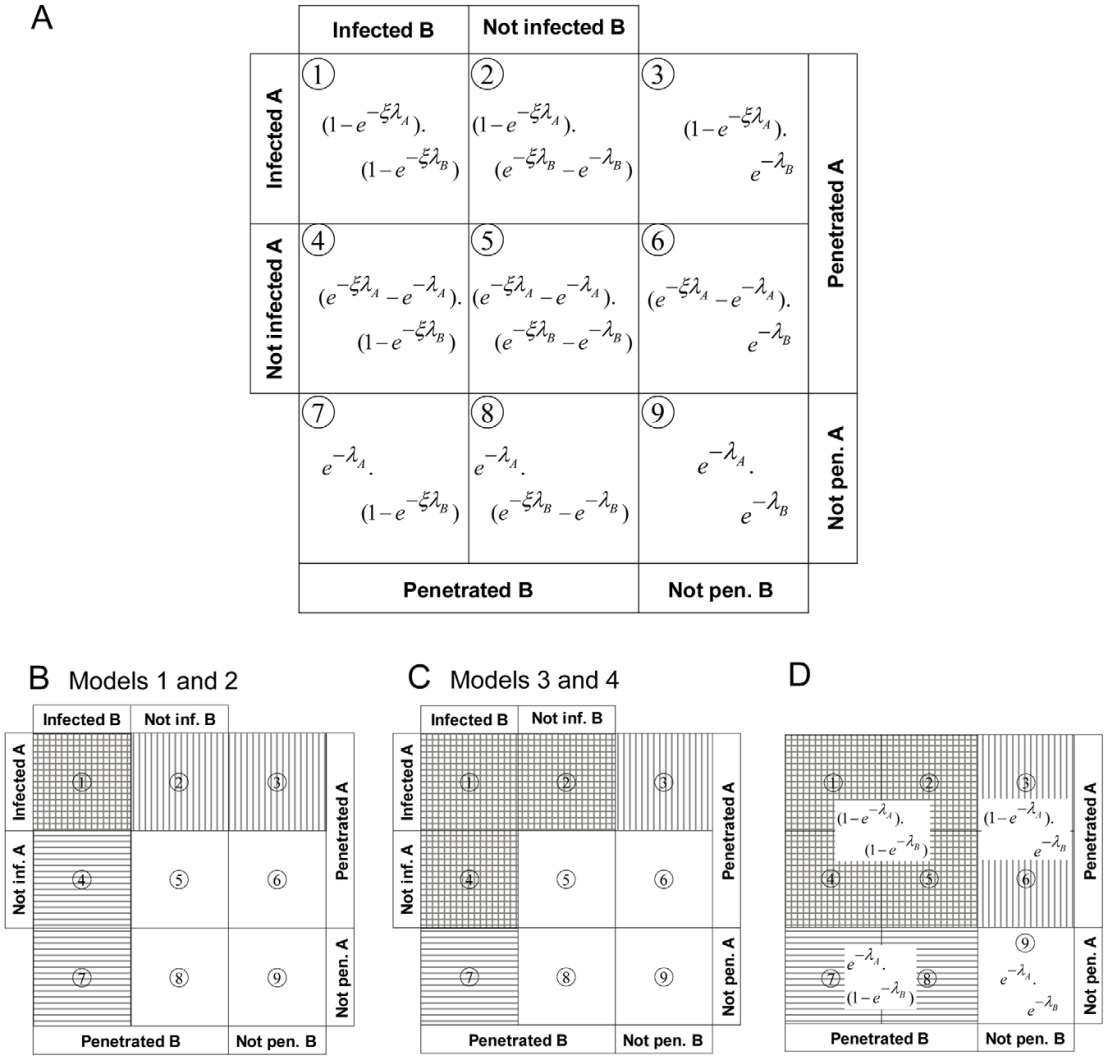
A diagrammatic representation of models presented in this paper. Diagram summarizing the expected proportions of hosts penetrated (or not) by genotypes A and/or B, and infected (or not) by genotypes A and/or B of a micro-parasite. The diagram is conditional on realizations of *ξ*, *λ*_A_ and *λ*_B_. In a real population of insects, there will be heterogeneity in these parameters, which will distort the proportionalities in the figure. Hence, the panel illustrates only the aspect of dependence, given penetration and infection chances, and it does not represent the effects of heterogeneity. Panel A gives an overview of the all possible outcomes – in terms of penetration and infection – after challenge of hosts with virions of genotypes A and B. Hosts can be penetrated or not, and if hosts have been penetrated, they can be infected. These outcomes hold for all four models presented here. Panel B corresponds to Models 1 and 2, where only infecting genotypes are genetically represented in the viral progeny. The expected fractions of alive larvae (no fill), penetrated by virions of genotype A only or penetrated by both genotypes and infected by A (vertically dashed), penetrated by genotype B only or penetrated by both genotypes and infected by B (horizontally dashed), and penetrated by both genotypes and infected by both genotypes (horizontally and vertically dashed) are illustrated. Panel C corresponds to Models 3 and 4, where all penetrating genotypes are genetically represented in the viral progeny, conditional upon the host being infected by (at least) one virion. The expected fractions of alive larvae (no fill), penetrated by genotype A only and infected by A (vertically dashed), penetrated by genotype B only and infected by B (horizontally dashed), and penetrated and infected by both genotypes or penetrated by both genotypes and infected by genotype A or penetrated by both genotypes and infected by genotype B (horizontally and vertically dashed). Note that In Models 1 and 2 (Panel B) the fraction of non-infected hosts conditional upon *ξ* is greater than the fraction non-penetrated, whereas in Models 3 and 4 (Panel C) that fraction exactly equals the fraction non-penetrated. Panel D represents the original, single-phase IAH model, where penetration of the host automatically leads to infection. The equations indicate the cumulative probabilities underlying boxes (e.g., 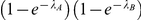 is the sum of boxes 1,2,4 and 5).

All four proposed models have in common that infection of both genotypes A and B is a two-phase process (Figure 2), i.e. penetration followed by successful infection results in the death of the host insect. For models 1 and 3, the probability that a virion causes infection (*ξ*), which is conditional upon the virion penetrating the host, is fixed. For models 2 and 4, *ξ* follows a beta distribution. The difference between models 1 and 2 on the one hand, and models 3 and 4 on the other hand, is that penetrated virions of both genotypes are not necessarily genetically represented in the viral progeny in models 1 and 2, but they are genetically represented in the progeny in models 3 and 4. Therewith, the probabilities (i) *P*(Ā ∩ B) (A absent, B present in PCR), (ii) *P*(A ∩ 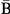) (A present, B absent in PCR), and (iii) *P*(A ∩ B) (both present in PCR) are affected (Figure 2 panels A and C). For given penetration chances *p*_A_ and *p*_B_ and infection probability *ξ*, the proportions of insects that are penetrated and infected by either or both virus types can be calculated, enabling a classification of insects into categories such as “penetrated by A, infected by B” and “penetrated by A, not penetrated by B”, etc. In total there are nine such categories, resulting from the 3*3 product of three states for each of two viruses: not penetrated, penetrated but not infected, and infected (Figure 2). Depending upon the postulated model these nine categories collapse to four categories that can be identified with PCR.

Model 1 is a special case of model 2. Model 1 is, as mentioned earlier, identical to the IAH model previously formulated, although it incorporates two infection phases resulting in a different parameterization with mean number of infecting virions *ξ⋅λ* (compare panel B and D of Figure 2). For model 1, *ξ* is fixed. The *ξ* values of less than one reduce the levels of host infection and mixed-genotype infection (population probabilities and probabilities for progeny of 1 host are equal) compared to the IAH model previously formulated [13]. In model 2, the probabilities apply to individual insect hosts for each different realization of *ξ*, and for populations they are conditional upon *ξ* being a realization from the beta distribution. To obtain the overall penetration and infection probabilities for model 2 at the level of the population of the host, we have to multiply the penetration probability *p* with the infection probability *ξ*, weighed by its beta density, and integrate over the domain of *ξ*: from zero to one.

Model 3 is a special case of model 4: *ξ* is fixed in model 3 and beta-distributed in model 4. We therefore provide only a detailed derivation of model 4. Conditional upon a realization *ξ* that is both independent for each virion and equal for all virions that have penetrated a particular host, the number of penetrating virions that infect the host - represented as the stochastic variable Ω - is Poisson distributed. A realization *ω* of this number, given the fixed chance in a host of *ξ* and the total number *n* of penetrating virions of genotypes A and B, is:
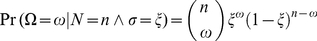
(3)


However, we want the unconditional probability of the number of penetrating virions that also infect the host and are therefore genetically represented in the viral progeny. Therefore, we first include that the realization *n* comes from a Poisson distribution by using Bayes' rule and sum over all possible values for *n*:
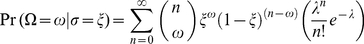
(4)where *λ* = *λ*_A_+*λ*_B_. It is easily shown that 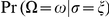 follows a Poisson distribution with mean *ξ⋅λ*.
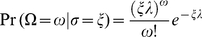
(5)


Above, *ξ* is for each host individual a random realization of the beta distribution:
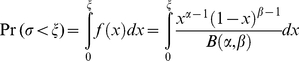
(6)


Here *α* and *β* are the two parameters that determine the shape of the beta distribution and *B*(*α*, *β*) is the beta function. The mean probability that a penetrating virion also infects the host is E(*σ*) = *α*/(*α*+*β*). From (5) and (6) we derive:
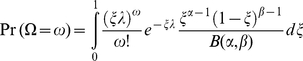
(7)


Hosts can survive if no virions penetrate the host or if virions did penetrate the host but were subsequently unsuccessful in bringing about host infection. Host mortality *(M*) is therefore one minus the zero-term (Pr(*ω* = 0)) of the Poisson distribution for the number of penetrating virions successfully causing host infection (i.e. subtracting the proportion hosts surviving from the full host population at risk):
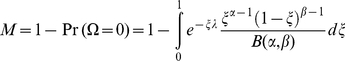
(8)


For the full host population, the probability for the number of infecting virions to be *ω* conditional upon the fixed chance *ξ* in a host is:
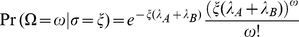
(9)


Thus the total fraction of hosts that becomes infected and dies given *ξ* (i.e. the sum of the boxes (1), (2), (3), (4) and (7) in Figure 2C) is:
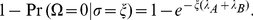
(10)


For penetration with *k* virions of genotype A and no virions of genotype B, given the fixed chance *ξ* of infecting the host for each virion, the probability that Ω virions infect is:
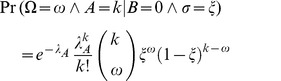
(11)


Using Bayes' rule and summing over all different realizations of *k* yields that 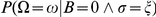 follows a Poisson distribution with mean 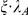. Therefore, the formula for the fraction dead in the “only penetrated by genotype A” class as represented by box (3) in Figure 2C is:
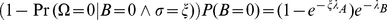
(12)


Because of the fact that genotype A and genotype B occur symmetrically in the formula, box (7) has for the fraction dead in the “only penetrated by genotype B” class 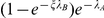. The formula for box (1) is the probability of being infected by A multiplied by the probability of being infected by B:
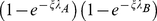
(13)


### Calibration of models

As data we used the absolute frequencies of death at seven doses, and the absolute frequencies of single genotype (A alone, and B alone) and mixed-genotype (A and B) infection at four doses, where intermediate to high mortality occurred (these doses thus are a subset of the seven doses used for dose response function), where frequency is measured as number of hosts with the described viral genotype(s). Calibration of the models was conducted in MatLab R2006b (Mathworks Inc.; Natick, MA), by minimizing the negative log likelihoods (NLL) with the Nelder Mead simplex direct search method.

The likelihoods were based on the multinomial distribution, because in the experimental set-up four different outcomes are possible. These are (1) host is healthy and no pathogen DNA can be detected, (2) host is dead and only genotype A is detected, (3) host is dead and only genotype B is detected, and (4) host is dead and both genotypes are detected. These four possibilities are mutually exclusive and together represent the full set of outcomes. Therefore, under the null hypothesis of independent action the number of occurrences of these four stochastic variables (*X*_1_, *X*_2_, *X*_3_, *X*_4_) follow a multinomial distribution with probabilities *p*_1_, …, *p*_4_ (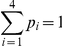). Thus the multinomial probability of a particular realization (*q*_1_, *q*_2_, *q*_3_, *q*_4_) is given by:
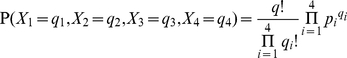
(14)


Note that 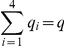 is the number of challenged insects. For our data, where only a fraction of the cadavers was processed using PCR, the multinomial distribution could not be applied directly to calculate the likelihood of the data under the different models. Therefore, we used a two-step procedure: first, the mortality response data for the whole tested population was used (eqn. 15) and subsequently the genotype presence data for the cadavers in the subsample (eqn. 16). As is clear from the description above the probability to survive a challenge at a certain dose is *p*_1_. Thus, the likelihood of the mortality data at each dose is:
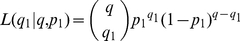
(15)


The likelihoods of the genotype presence in the subsample of 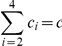 cadavers (*c*<*q*) was calculated using:
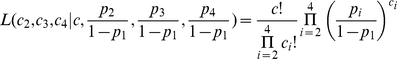
(16)


Given the data we calculated seven negative log-likelihood values for the mortality response and four for the genotype response. These were all summed to obtain the overall negative log-likelihood, which could then be minimized.

In the function defining the mathematical model for the data, calculation of population level probabilities was achieved by numerical integration of model equations over the probability distribution of 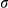 that characterizes the variability in host susceptibility. Integrals were calculated numerically using a recursive adaptive higher order integration method, as implemented in the function QUADL in MatLab R2006b. All results of numerical integrations over the beta probability density function (PDF) were verified by stochastic simulations of the infection process, based on sufficiently large numbers of random draws to average out the variability. Results of parameter optimizations were corroborated by grid searches in MatLab. Thus we made sure that local minima were avoided in the parameter estimation.

## Results

### Challenge of *S. exigua* L5 larvae with AcMNPV leads to a shallower dose response than predicted by IAH

**Figure 3:**
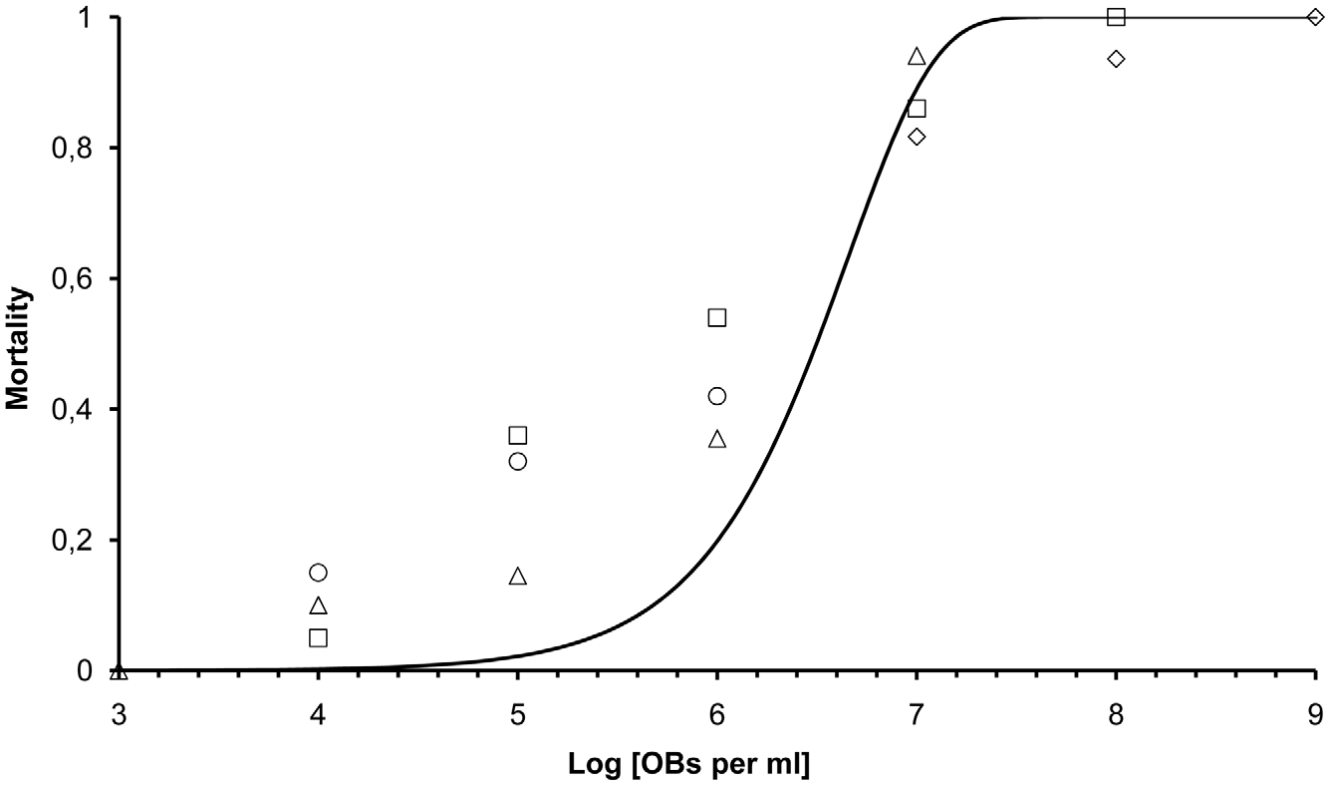
Dose response for AcMNPV infection of *S. exigua* L5. On the x-axis is the log10 of the virus dose (OBs per ml) droplet fed to larvae. The proportion of hosts dying is on the y-axis. The different symbols represent different replicates, which covered different ranges in dose. The line represents the IAH dose response relationship by non-linear regression (mortality  = 1 - exp(-*p*· OB concentration), SPSS 15.0), rendering *p* = 2.205×10^−7^.

The dose response for AcMNPV infection of *S. exigua* L5 was first determined (Figure 3). Previous reports suggested that dose response in L5 larvae of this insect was shallow [13], [42], [43]. Confirming these previous reports, we found a dose response that was much shallower than predicted by a model based on IAH and fixed probability of infection among hosts.

### Frequency of mixed-genotype infections increases with dose and is higher than IAH predictions

**Figure 4:**
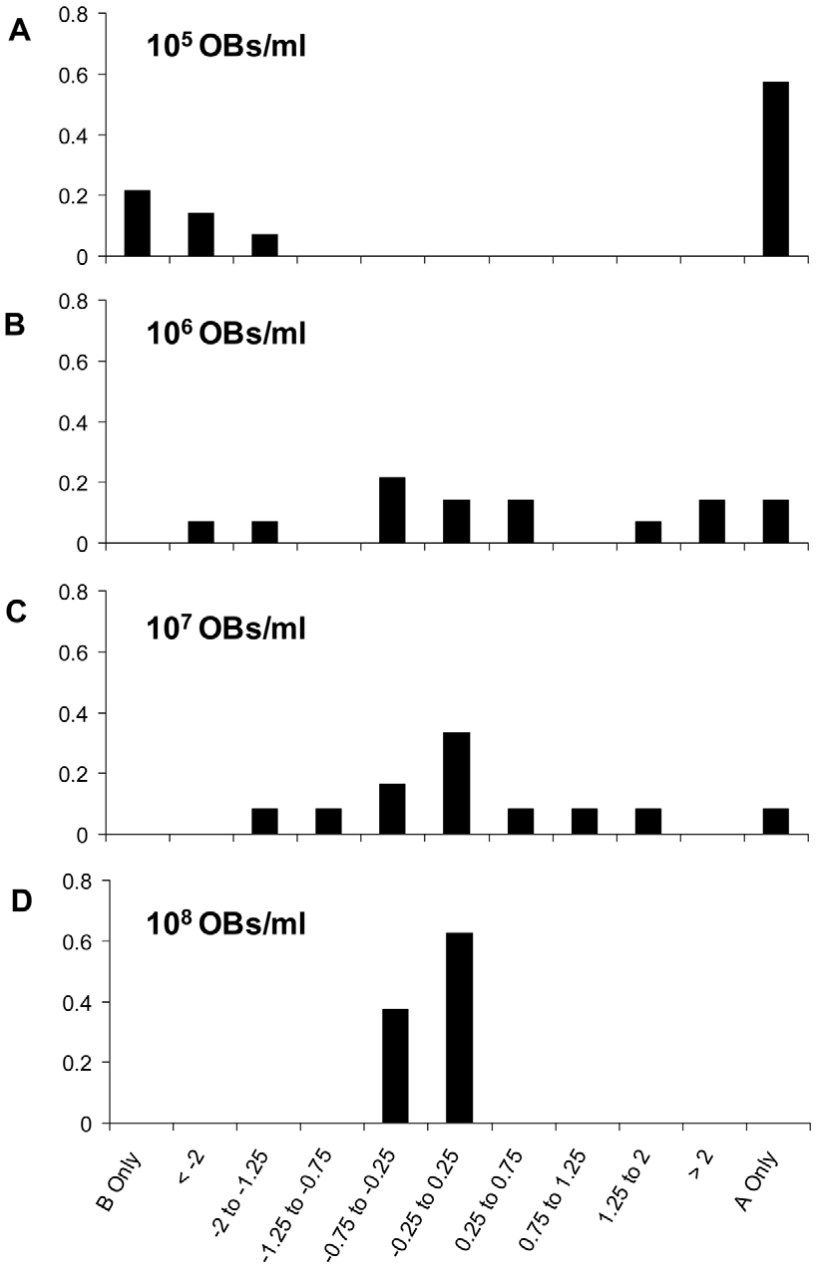
Ratio of genotype A to genotype B in infected hosts. The log-transformed genotype ratio (A:B) is given for *S. exigua* L5 larvae at different inoculum OB concentrations, as indicated in the upper left-hand corner of each panel. On the x-axis is the log10 of the genotype ratio (A:B), and on the y-axis frequency. Host survival *(S)* decreased with inoculum OB concentration, from *S* = 0.72 (10^5^ OBs per ml) to *S* = 0.03 (10^8^ OBs per ml). The number of hosts per dose are 14 (10^5^ and 10^6^ OBs per ml), 12 (10^7^ OBs per ml) and 8 (10^8^ OBs per ml).

The frequency of genotypes A and B was determined at different doses using qPCR (Table 2). Using previously described methods [13], we compared IAH predictions of the frequency of mixed-genotype infection, *P'*(A ∩ B), to the data (Table 2). The frequency of mixed-genotype infections was significantly higher than predicted by IAH at all doses except the lowest (10^5^ OBs/ml). At the lowest dose, there were mostly single-genotype infections (∼80%). At the higher doses (≥10^6^ OBs/ml), mixed-genotype infections predominated. These data are therefore in agreement with our previous findings for *S. exigua* L5, where we rejected an IAH-based model [13]. The genotype ratio data (Figure 4) show that as dose and mortality increased, the variation in genotype ratio also decreased. Levene's test demonstrated that variances of the log-transformed genotype ratios were not homogenous for different doses, both for all data (Levene statistic  = 25.534; degrees of freedom  = 3, 44; *P*<0.001) and for only the co-infected samples of the highest three doses (Levene statistic  = 4.571; degrees of freedom  = 2, 28; *P* = 0.019). The decrease in variation with increasing dose likely results from an increase in the number of infecting virions.

**Table 2:** Frequency of mixed-genotype infection at different doses.

Dose (OBs/ml)	Host survival	Mixed/Total	*f*(A∩B)	*P*'(A∩B)	Significance
10^5^	0.72	3/14	0.214	0.076	0.085
10^6^	0.55	12/14	0.857	0.138	<0.001***
10^7^	0.13	11/12	0.917	0.440	0.001**
10^8^	0.03	8/8	1.000	0.667	0.039*

The number of mixed-genotype infected larvae and the total number of larvae tested by qPCR (mixed/total), observed frequency of mixed-genotype infection (*f*(A∩B)), IAH predicted frequency of mixed-genotype infection (*P'*(A∩B)), and the significance level of a one-sided binomial test comparing *f*(A∩B) and *P'*(A∩B) (significance) are given. * indicates statistical significance at a 0.05 significance threshold, ** at a 0.01 threshold, and *** at a 0.001 threshold.

### Support for an IAH-based model assuming heterogeneous host susceptibility

**Figure 5:**
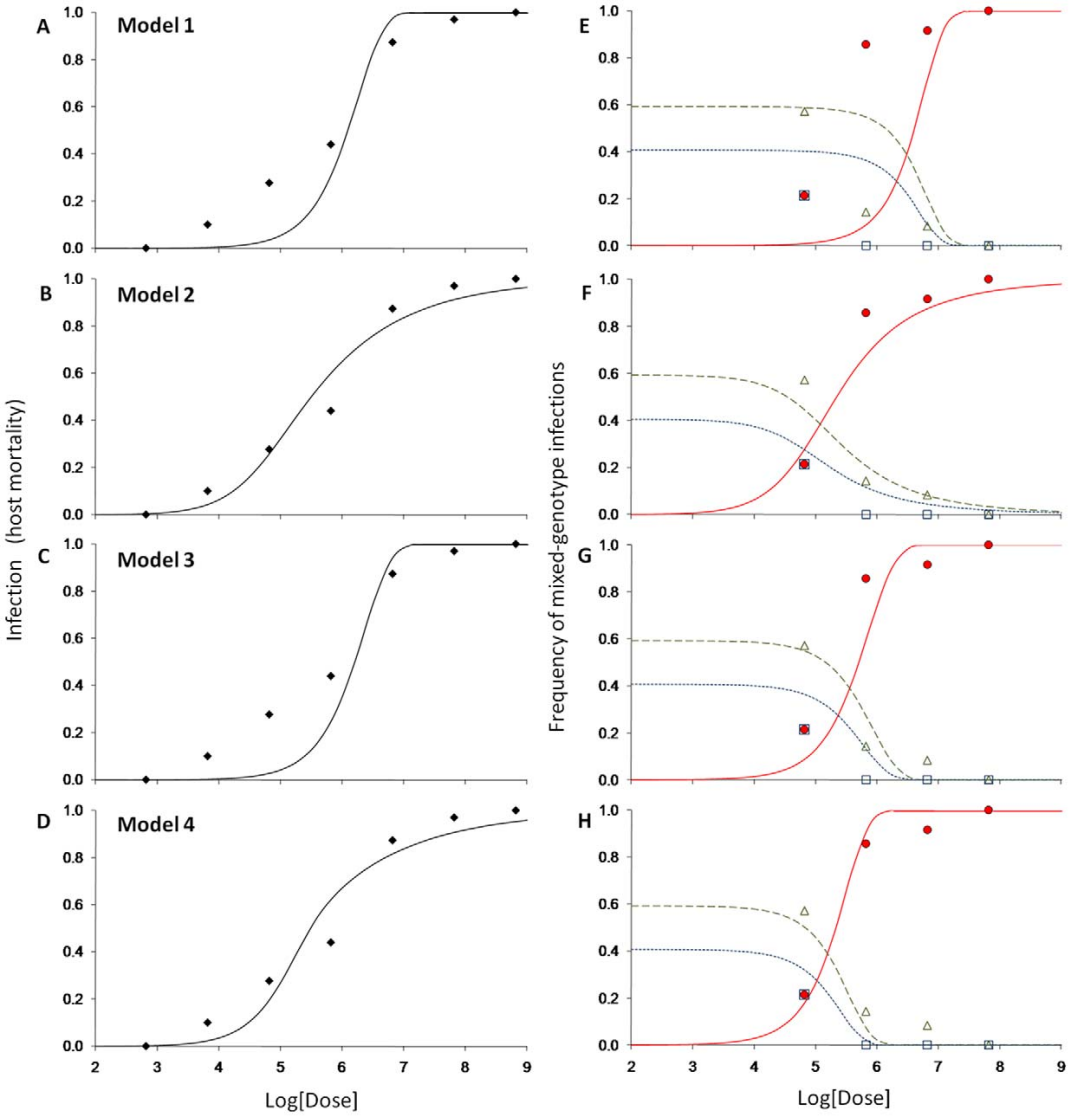
Fitted models and experimental data. Fitted Models 1–4 compared with experimental data. In all panels, the log of the dose is on the x-axis and frequency on the y-axis. Dose mortality responses are given in the left-hand panels (A–D). Here, diamonds are the experimental data and the lines model predictions. Relationships between dose and rate of mixed-genotype infection in cadavers are presented in the right hand panels (E–H). Again, markers denote experimental data while lines denote model predictions: red circles and the red solid line denote mixed infection with genotypes A and B, green triangles and the green dotted line denote infections with genotype A only, and blue squares and the fine dotted blue line denote infections with genotypes B only. Horizontally adjacent panels pertain to the same model, as indicated in the left hand panel (e.g., panels A and E correspond to model 1). Model 2 gives the best description of the data (see Table 3).

**Figure 6:**
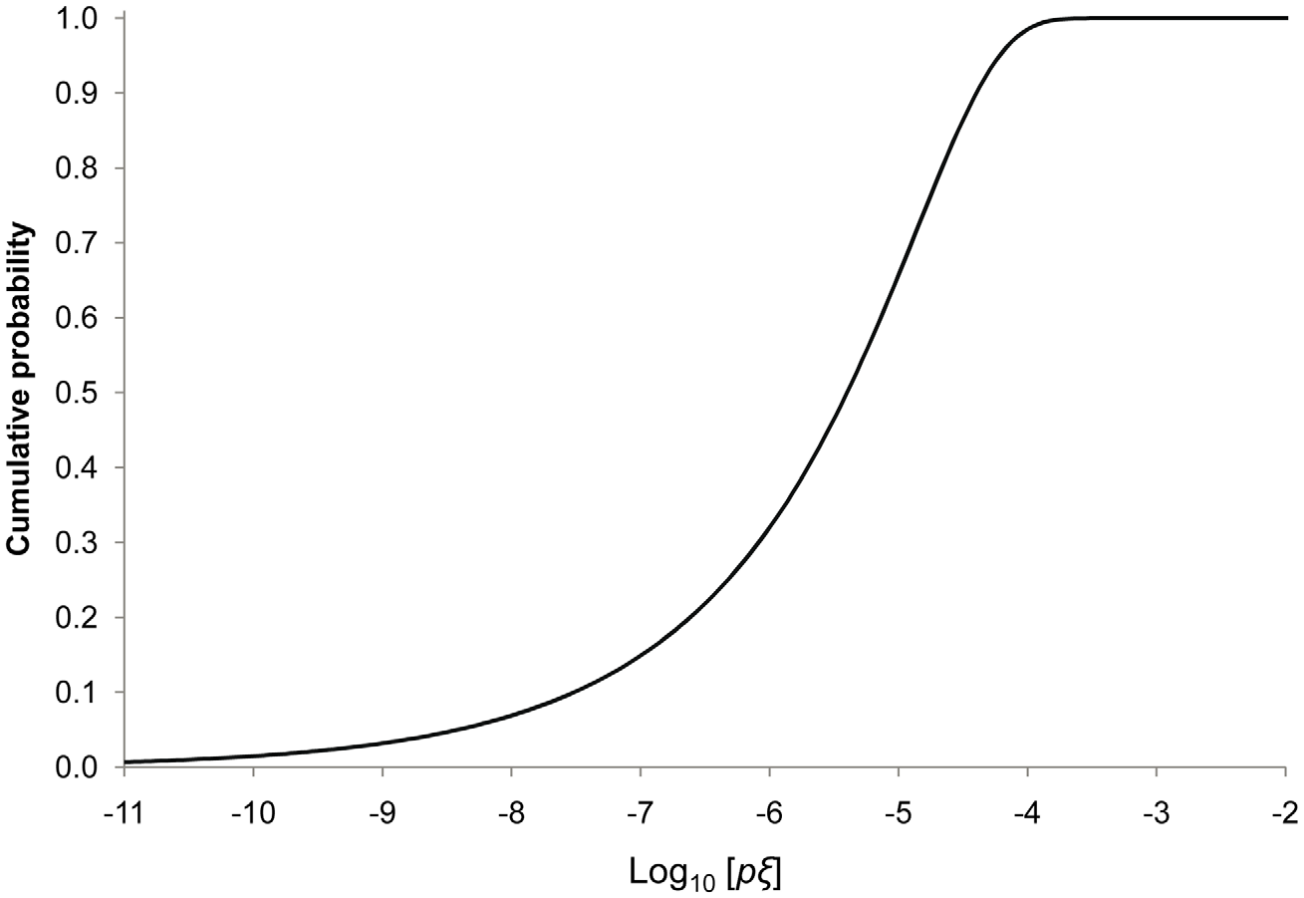
The predicted distribution of host susceptibility. The distribution of host susceptibility predicted by model 2 with fitted parameters: *p* = 0.0059, *α* = 0.335, *β* = 143 (Table 3). Host susceptibility is characterized by the overall probability of disease causation for a single ODV, *pξ*, which is the product of the *per virion* chances of successful penetration, *p*, and the probability of successful infection given successful penetration, *ξ*. The shape of the cumulative distribution of *pξ* is the same as that of the beta distribution for *ξ*, but the domain of the former is limited to (0, *p*], and the distribution is accordingly shifted to the left compared to the beta distribution for *ξ w*hich has (0,1] as its domain. A logarithmic scale is used for the abscissa to represent the broad range of susceptibility in the host population, which is reflected in shallow dose response and is – as shown in this paper – intrinsically associated with a high incidence of mixed genotypes in cadavers.

The four models were calibrated (Table 3) and the solutions were plotted with the data (Figure 5). Models 1 (independent action, no heterogeneity; ΔAIC = 209.4) and 3 (facilitation, no heterogeneity; ΔAIC = 157.2) gave the poorest fits; the modeled dose response relationships were steeper than the data due to the absence of variation in host susceptibility. Model 3, which incorporates the idea that all penetrating virions contribute to infection, provided a better description of the frequency of mixed-genotype infection than model 1, although there was clear lack of fit (Figure 5). Models 2 and 4 gave better fits than models 1 and 3: dose response and frequency of mixed-genotype were much closer to the data. There was, however, clearly more support for model 2 (independent action, heterogeneity) than for Model 4 (facilitation, heterogeneity; ΔAIC = 25.4). Note that model 2 is not a special case of model 4. In model 2 penetrating virions have a chance of infecting the host; in model 4 all penetrating virions are genetically represented if the host becomes infected. In Figure 6, we plot the cumulative distribution of host susceptibility, characterized by the product of penetration probability *p* (0.0059) and infection probability *ξ*, with mean *ξ = α/(α*+*β*) = 0.0023 and scale parameter *α*+*β* = 143.63. The resulting distribution of *pξ* has the same shape as the beta distribution for *α* = 0.335 and *β* = 143; however, the domain is constrained to (0, *p*), and the resulting cumulative density is accordingly shifted to the left compared to the distribution of *ξ*.

**Table 3:** AIC and estimated parameter table.

Model	NLL	Parameters	AIC	ΔAIC	*p*	*α*	*β*	
2	27.2	3	60.4		0.0059	0.335	143	–
4	39.9	3	85.8	25.4	1.76 10^−5^	0.300	0.458	–
3	106.8	2	217.6	157.2	5.8 10^−6^	–	–	*ξ* = 0.14
1	133.9	1	269.8	209.4	–	–	–	*pξ* = 1.05 10^−6^

For the calibrated models, the negative log likelihood (NLL), the number of model parameters are given, the Akaike information criterion (AIC), and the difference in AIC between a model and the best fitting model (ΔAIC), model 2, are given.

### Generalization with Jensen's inequality

We will first illustrate with a numerical example that heterogeneous host susceptibility must necessarily result in enhanced prevalence of mixed pathogen genotypes in infected hosts. Next, we generalize the example and give some further theoretical and general considerations.

**Figure 7:**
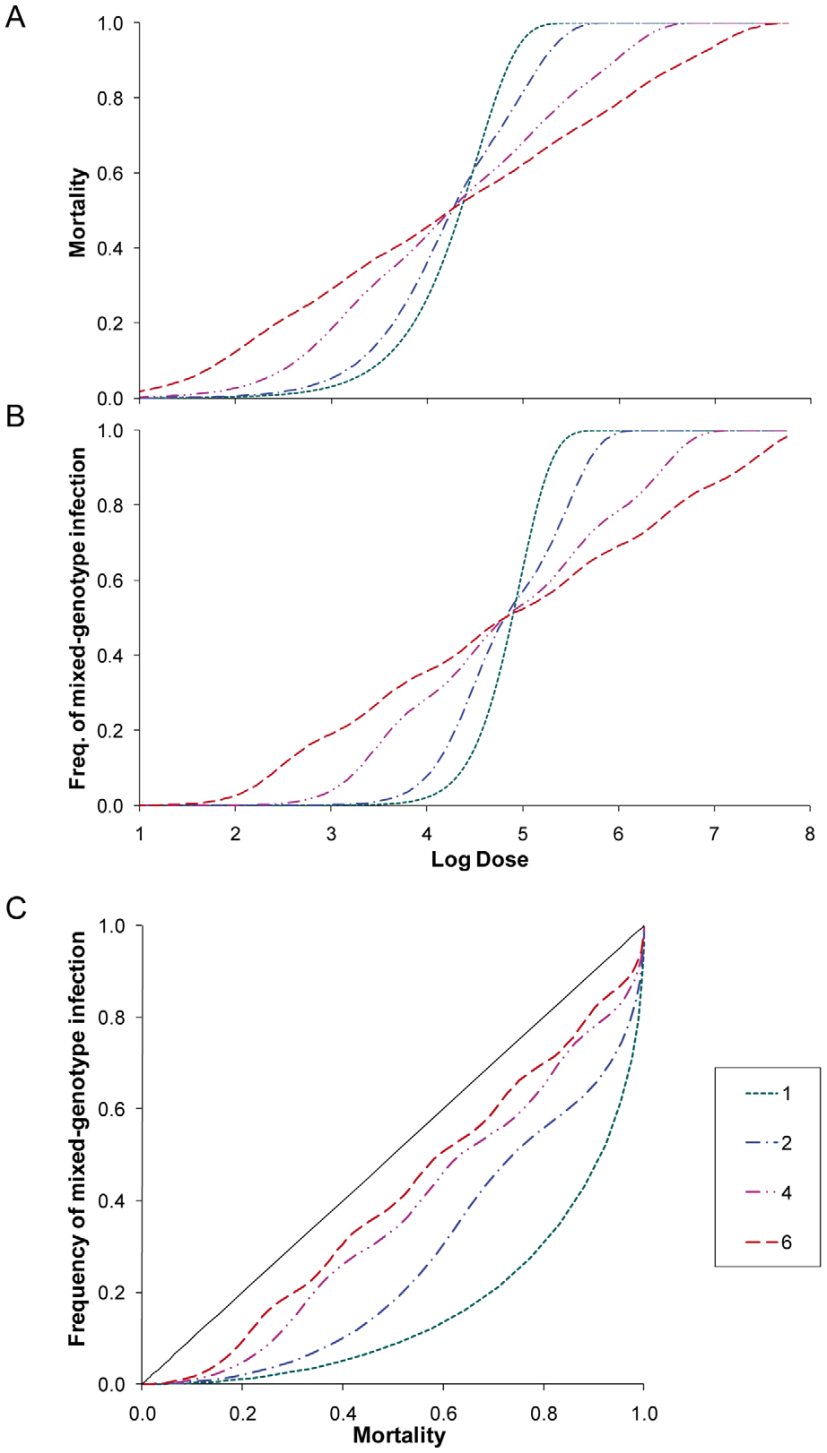
The effects of heterogeneity in host susceptibility. We assume a host population that is composed of 1, 2, 4 or 6 classes of individuals, varying in their susceptibility to a virus. The number of classes is given in the legend, and applies to all three panels. (E.g. A value of ‘1’ indicates the infection probabilities are the same for all hosts, so in this instance the host population is homogeneous. ‘6’ indicates that there are six host classes with different susceptibilities to the virus) The following infection probabilities were assumed: 1 class, 10^−4.5^; 2 classes, 10^−4^, 10^−5^; 4 classes, 10^−3^, 10^−4^; 10^−5^, 10^−6^; 6 classes, 10^−2^, 10^−3^, 10^−4^; 10^−5^, 10^−6^, 10^−7^. The geometric mean infection probability is 10^−4.5^ in all four cases. A virus population composed of two genotypes in a 1∶1 mixture, and no differences in infection probability for these genotypes, was assumed. In Panel A, the dose response relationship is illustrated. On the x-axis is the log of dose, and on the y-axis mortality. Note that as more host classes are introduced, the dose response relationship becomes shallower. In Panel B is the frequency of mixed-genotype infection, which follows a similar trend with dose. Panel C is the relationship between host mortality (x-axis) and the frequency of mixed-genotype infection (y-axis). The solid line is a 1∶1 relationship between mortality and the frequency of mixed-genotype infection. As a more heterogeneity is introduced in the host population, the frequency of mixed-genotype infection becomes higher and eventually approaches the 1∶1 line (both micro-parasite genotypes are established in all hosts at any level of mortality).

For sake of argument, consider a hypothetical situation similar to our experimental setup: hosts are challenged with a virus population made up of two genotypes. The host population is assumed to be heterogeneous with respect to virus susceptibility: half of the host population is susceptible and half is resistant. Under IAH, the effects of host heterogeneity on dose response and the frequency of mixed-genotype infection can be predicted. This can be done simply by considering the susceptible and resistant host populations separately, and subsequently combining model predictions (e.g. mortality in susceptible hosts and mortality in resistant hosts). The shapes of both dose response and rate of mixed-genotype infection are then shallower (Figure 7A and B). If for these same instances we plot mortality vs. the frequency of mixed-genotype infection, we find that variability in host susceptibility results in a higher frequency of mixed-genotype infection at all levels of mortality (Figure 7C). For a somewhat more realistic illustration, the host population is subsequently subdivided into a greater number of classes of susceptibility, covering a logarithmic series for the susceptibility parameter. The dose response relationship (Figure 7A), and dose mixed-genotype infection (Figure 7B) become shallower as heterogeneity in the host population increases. The relationship between mortality and mixed-genotype infection (Figure 7C) approaches the line where all infections are mixed-genotype infections as host heterogeneity is increased. In the final section of the results we show mathematically that heterogeneous host susceptibility necessarily leads to an equal or higher frequency of mixed genotype infections than for the IAH model, generalizing the principle illustrated by example above.

Here we show in a more general case that, at any given level of host mortality, the proportion of mixed-genotype infections under the IAH model with a variable infection chance necessarily equals or exceeds the proportion of mixed-genotype infections under IAH and a fixed infection chance. Consider the convex relationship between *x_ρ_*(*n*) and *y_ρ_*(*n*) where *x_ρ_*(*n*) is the expected level of host mortality at dose *n* and fixed infection chance *ρ* and *y_ρ_*(*n*) is the corresponding proportion of mixed infections. For the special case that two virus genotypes A and B have identical infection chance, and are equally represented in the inoculum, it can be shown that the relationship between *y* and *x* is given by:
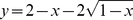
(17)where subscript *ρ* and argument *n* are dropped for clarity. This is a convex relationship. For the more general case that the proportion of genotypes in the inoculum is *f*_A_ and *f*_B_ we get:
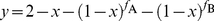
(18)which is also a convex relationship, unless either *f*_A_ or *f*_B_ is zero, in which case mixed-genotype infection is impossible. In all cases, the relationship between *y_ρ_*(*N*) and *x_ρ_*(*N*) in a homogeneous population with a single value of *ρ* is convex. In a heterogeneous population, there is representation of larvae with different value of *ρ* and the population response is a weighted average of point pairs (*x_ρ_*(*n*), *y_ρ_*(*n*)) over different values of *ρ*. Based on Jensen's inequality, which states that for a real convex function *φ*, numbers *x_i_* in its domain, and positive weights *a_i_*:
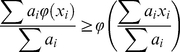
(19)


We know that this averaging must result in a population response that is to the left or above the convex relationship relating *y_ρ_*(*n*) and *x_ρ_*(*n*) in a homogeneous population, completing the argument.

## Discussion

There have been lingering doubts about the applicability of IAH and the reasons why this model is so often in disagreement with data [7], [9], [13]. We previously considered whether IAH can describe dose response and the frequency of mixed-genotype infection in the baculovirus-insect larvae pathosystem [13]. IAH was not supported in four out of six pathosystems tested, raising an intriguing question: how are a shallow dose response and a high frequency of mixed-genotype infection to be simultaneously explained in a single model? Here we examined the infection process of AcMNPV in *S. exigua* L5 in detail, and considered a number of models that could potentially describe the infection process when IAH fails. The different modifications made to this model are all plausible given the current understanding of baculovirus biology (see methods for a motivation of each model). The mechanisms we considered were (i) differences in host susceptibility, (ii) dependence, specifically facilitation in the infection process: if one virion infects the host (e.g., breaks the host systemic defense), then all penetrating virions are represented in the viral progeny generated and detected by qPCR, and (iii) a combination of these two mechanisms.

We found that the single assumption of heterogeneous host susceptibility was sufficient to describe the infection process (Figure 5 and Table 3). It has been suggested this mechanism accounts for the shallow dose response observed for baculoviruses, in particular fourth or fifth-instar larvae [6], [11], [37]. Others have suggested the same mechanism for understanding dose response in other pathosystems [7], [9], [12]. Moreover, there is good evidence that heterogeneous host susceptibility leads to deviations from mass-action predictions of transmission for baculoviruses [6], [44] and for bacterial micro-parasites of protozoans [45]. Here we show that the assumption of heterogeneous host susceptibility also helps reconcile the principle of independent action with a high frequency of mixed-genotype infection.

Our results show that independent action may very well apply in those instances where we previously rejected an IAH-based model with a constant probability of host infection [13]. The principle of independent action still applies, however, because variation in host susceptibility leads to a higher frequency of mixed-genotype establishment. In our previous work, the frequency of mixed-genotype infection was consistently higher than model predictions, although the differences were not always significant [13]. As there will always be minor differences in host susceptibility in any experimental setting, a departure from the IAH model with fixed probability of infection would be expected in many cases, that is: the null model of no variability is often too naive. However, the differences in host susceptibility are in this particular case not minor; estimated model 2 parameters predict a broad range of susceptibility to AcMNPV in the host population (Figure 6).

This conclusion, however, raises the question of why there would be greater differences in host susceptibility in later (L5) as compared to earlier (L3) larval stages of the Lepidopteran insects that we used in our experiments: *S. exigua* (this study and [8]) and two other species: cabbage looper, *Trichoplusia ni*, and cabbage moth, *Mamestra brassicae*. One reason may be that larval resistance to baculoviruses has a high phenotypic plasticity [46], [47]. Therefore, we speculate that as development progresses, variability in susceptibility will also increase. Resistance to pathogens is linked to melanization [47], meaning that it might be possible to select a more homogeneous insect population, with respect to viral resistance, based on visual characteristics of the larvae. Another reason for greater variability in susceptibility in later larval stages is that intrastadial developmental resistance (IDR) - an increase in host resistance within an instar [48], [49] – may arise quicker in later larval instars. If larvae were selected within a smaller time window (e.g., 2 h instead of 8 h), we expect the data to be more similar to predictions from the IAH model with a fixed probability of infection.

In this specific instance there is in principle no reason to reject IAH. However, our results support the hypothesis that differences in host susceptibility lead to rejection of the simplest of infection models: IAH with a constant infection probability. Shallow dose response relationships are often observed in laboratory experiments [9], [11], [13], but it has not been possible to convincingly determine whether a shallow dose response arises due to variation in host susceptibility or dependent action [7], [9]. Our results demonstrate that the assumption of variation in host susceptibility is sufficient to describe the infection process, suggesting that departures from IAH need not be invoked as an explanation. The generality of this conclusion is a key issue, because IAH, as one of the cornerstones of the mass-action principle, pervades models of disease ecology. Our results therefore reinforce the importance of incorporating differences in host susceptibility in models of infectious disease ecology [6], [9], such as age-based risk structure [4], [50]. Moreover, our results provide further support for using experimental estimates of heterogeneity in host susceptibility obtained from dose response relationships [6], [38], [51], and suggest that most deviations from IAH predictions may be caused by variation in host susceptibility. No additional mechanisms need to be invoked to explain the data.

## References

[1] Anderson RM, May RM (1979). Population biology of infectious disease 1.. Nature.

[2] Anderson RM, May RM (1982). Directly transmitted infectious diseases: control by vaccination.. Science.

[3] Nesse RM, Stearns SC (2008). The great opportunity: Evolutionary applications to medicine and public health.. Evol Appl.

[4] Keeling MJ, Rohani P (2008). Modelling infectious diseases in humans and animals..

[5] McCallum H, Barlow N, Hone J (2001). How should pathogen transmission be modelled?. Trends Ecol Evol.

[6] Dwyer G, Elkinton JS, Buonaccorsi JP (1997). Host heterogeneity in susceptibility and disease dynamics: Tests of a mathematical model.. Am Nat.

[7] Regoes RR, Hottinger JW, Sygnarski L, Ebert D (2003). The infection rate of *Daphnia magna* by *Pasteuria ramosa* conforms with the mass-action principle.. Epidemiol Infect.

[8] Teunis PFM, Havelaar AH (2000). The Beta Poisson dose-response model is not a single-hit model.. Risk Anal.

[9] Ben-Ami F, Regoes RR, Ebert D (2008). A quantitative test of the relationship between parasite dose and infection probability across different host–parasite combinations.. Proc R Soc B.

[10] Dieu BTM, Zwart MP, Vlak JM (2010). Can VNTRs be used to study genetic variation within white spot syndrome virus isolates?. J Fish Dis.

[11] Ridout MS, Fenlon JS, Hughes PR (1993). A generalized one-hit model for bioassays of insect viruses.. Biometrics.

[12] Teunis PFM, Moe CL, Liu P, Miller SE, Lindesmith L (2008). Norwalk virus: How infectious is it?. J Med Virol.

[13] Zwart MP, Hemerik L, Cory JS, de Visser JAGM, Bianchi FJJA (2009). An experimental test of the independent action hypothesis in virus-insect pathosystems.. Proc R Soc B.

[14] Taylor LH, Walliker D, Read AF (1997). Mixed-genotype infections of the rodent malaria *Plasmodium chabaudi* are more infectious to mosquitoes than single-genotype infections.. Parasitology.

[15] Vignuzzi M, Stone JK, Arnold JJ, Cameron CE, Andino R (2006). Quasispecies diversity determines pathogenesis through cooperative interactions in a viral population.. Nature.

[16] Simon O, Williams T, Caballero P, Lopez-Ferber M (2006). Dynamics of deletion genotypes in an experimental insect virus population.. Proc R Soc B.

[17] Smith IRL, Crook NE (1988). In vivo isolation of baculovirus genotypes.. Virology.

[18] Cory JS, Green BM, Paul RK, Hunter-Fujita F (2005). Genotypic and phenotypic diversity of a baculovirus population within an individual insect host.. J Invertebr Pathol.

[19] Simon O, Williams T, Lopez-Ferber M, Caballero P (2004). Genetic structure of a *Spodoptera frugiperda* nucleopolyhedrovirus population: High prevalence of deletion genotypes.. Appl Environ Microbiol.

[20] Ebert D, Weisser WW (1997). Optimal killing for obligate killers: the evolution of life histories and virulence of semelparous parasites.. Proc R Soc B:.

[21] Cory JS, Myers JH (2003). The ecology and evolution of insect baculoviruses.. Annu Rev Ecol Evol S.

[22] Hodgson DJ, Hitchman RB, Vanbergen AJ, Hails RS, Possee RD (2004). Host ecology determines the relative fitness of virus genotypes in mixed-genotype nucleopolyhedrovirus infections.. J Evol Biol.

[23] Zwart MP, van der Werf W, Georgievska L, van Oers MM, Vlak JM (2010). Mixed-genotype infections of *Trichoplusia ni* larvae with *Autographa californica* multicapsid nucleopolyhedrovirus: Speed of action and persistence of a recombinant in serial passage.. Biol Control.

[24] Zwart MP, van der Werf W, van Oers MM, Hemerik L, van Lent JMV (2009). Mixed infections and the competitive fitness of faster-acting genetically modified viruses.. Evol Appl.

[25] Georgievska L, Joosten N, Hoover K, Cory JS, Vlak JM (2010). Effects of single and mixed infections with wild type and genetically modified *Helicoverpa armigera* nucleopolyhedrovirus on movement behaviour of cotton bollworm larvae.. Entomol Exp Appl.

[26] Druett HA (1952). Bacterial invasion.. Nature.

[27] Peto S (1953). A dose-response equation for the invasion of micro-organisms.. Biometrics.

[28] Zwart MP, van Oers MA, Cory JS, van Lent JWM, van der Werf W (2008). Development of a quantitative real-time PCR for determination of genotype frequencies for studies in baculovirus population biology.. J Virol Methods.

[29] Meynell GG, Stocker BAD (1957). Some hypotheses on the aetiology of fatal infections in partially resistant hosts and their application to mice challenged with *Salmonella-paratyphi-B* or *Salmonella-typhimurium* by intraperitoneal injection.. J Gen Microbiol.

[30] Moxon ER, Murphy PA (1978). *Hemophilus influenzae* bacteremia and meningitis resulting from survival of a single organism.. Proc Natl Acad Sci USA.

[31] Clavijo G, Williams T, Munoz D, Caballero P, Lopez-Ferber M (2010). Mixed genotype transmission bodies and virions contribute to the maintenance of diversity in an insect virus.. Proc R Soc B.

[32] Ridout MS, Fenlon JS (1991). Analyzing dose-response data when doses are subject to error.. Ann Appl Biol.

[33] Sun XL, Wang HL, Sun XC, Chen XW, Peng CM (2004). Biological activity and field efficacy of a genetically modified *Helicoverpa armigera* single-nucleocapsid nucleopolyhedrovirus expressing an insect-selective toxin from a chimeric promoter.. Biol Control.

[34] Federici BA, Miller LK (1997). Baculovirus pathogenesis;.

[35] Myers JH, Malakar R, Cory JS (2000). Sublethal nucleopolyhedrovirus infection effects on female pupal weight, egg mass size, and vertical transmission in gypsy moth (Lepidoptera: Lymantriidae).. Environ Entomol.

[36] Sait SM, Begon M, Thompson DJ (1994). The effects of a sublethal baculovirus infection in the Indian meal moth, *Plodia interpunctella*.. J Anim Ecol.

[37] Bianchi F, van der Werf W, Vlak JM (2002). Validation of a comprehensive process-based model for the biological control of beet armyworm, *Spodoptera exigua*, with baculoviruses in greenhouses.. Biol Control.

[38] Ben-Ami F, Ebert D, Regoes RR (2010). Pathogen dose infectivity curves as a method to analyze the distribution of host susceptibility: a quantitative assessment of maternal effects after food stress and pathogen exposure.. Am Nat.

[39] Olkin I, Gleser, J L, Derman C (1994). Probability models and applications..

[40] Hughes DS, Possee RD, King LA (1993). Activation and detection of a latent baculovirus resembling *Mamestra brassicae* nuclear polyhedrosis virus in *M. brassicae* insects.. Virology.

[41] Jurkovicova M (1979). Activation of a latent virus infections in larvae of *Adoxophyes orana* (Lepidoptera, Tortricidae) and *Barathra brassicae* (Lepideoptera, Noctuidae) by foreign polyhedra.. J Invertebr Pathol.

[42] Bianchi F, Snoeijing I, van der Werf W, Mans RMW, Smits PH (2000). Biological activity of SeMNPV, AcMNPV, and three AcMNPV deletion mutants against *Spodoptera exigua* larvae (Lepidoptera: Noctuidae).. J Invertebr Pathol.

[43] Bianchi F, Vlak JM, Rabbinge R, Van der Werf W (2002). Biological control of beet armyworm, *Spodoptera exigua*, with baculoviruses in greenhouses: Development of a comprehensive process-based model.. Biol Control.

[44] D'Amico V, Elkinton JS, Dwyer G, Burand JP, Buonaccorsi JP (1996). Virus transmission in gypsy moths is not a simple mass action process.. Ecology.

[45] Fels D, Vignon M, Kaltz O (2008). Ecological and genetic determinants of multiple infection and aggregation in a microbial host-parasite system.. Parasitology.

[46] Goulson D, Cory JS (1995). Responses of *Mamestra brassicae* (Lepidoptera, Noctuidae) to crowding-interactions with disease resistance, color phase and growth.. Oecologia.

[47] Reeson AF, Wilson K, Gunn A, Hails RS, Goulson D (1998). Baculovirus resistance in the noctuid *Spodoptera exempta* is phenotypically plastic and responds to population density.. Proc R Soc B.

[48] Grove MJ, Hoover K (2007). Intrastadial developmental resistance of third instar gypsy moths (*Lymantria dispar* L.) to *L. dispar* nucleopolyhedrovirus.. Biol Control.

[49] Hoover K, Grove MJ, Su SZ (2002). Systemic component to intrastadial developmental resistance in *Lymantria dispar* to its baculovirus.. Biol Control.

[50] Diekmann O, Heesterbeek JAP, Leven S (2000). Mathematical epidemiology of infectious diseases;.

[51] Elderd BD, Dushoff J, Dwyer G (2008). Host-pathogen interactions, insect outbreaks, and natural selection for disease resistance.. Am Nat.

